# Comparison of usefulness between the Mini-Balance Evaluation Systems Test and the Berg Balance Scale for measuring balance in patients with subacute stroke: a prospective cohort study

**DOI:** 10.3389/fresc.2023.1308706

**Published:** 2024-01-04

**Authors:** Seigo Inoue, Hideyuki Takagi, Emiko Tan, Chisato Oyama, Eri Otaka, Kunitsugu Kondo, Yohei Otaka

**Affiliations:** ^1^ Department of Rehabilitation Medicine, Tokyo Bay Rehabilitation Hospital, Chiba, Japan; ^2^ Assistive Robot Center, National Center for Geriatrics and Gerontology, Aichi, Japan; ^3^ Department of Rehabilitation Medicine I, School of Medicine, Fujita Health University, Aichi, Japan

**Keywords:** balance, cerebrovascular disease, fall, rehabilitation, usefulness, responsiveness

## Abstract

**Introduction:**

The aim of this study was to compare the clinical applicability of the Mini-Balance Evaluation Systems Test and the Berg Balance Scale for measuring balance in inpatients with subacute stroke.

**Methods:**

This was a prospective observational study which included 58 consecutive patients admitted to a convalescent rehabilitation hospital with a first-ever stroke and who met the inclusion/exclusion criteria. The Mini-Balance Evaluation Systems Test and the Berg Balance Scale were used to assess patient balance at admission and discharge. The ceiling and floor effects and responsiveness of each balance score were examined. In addition, receiver operating characteristic analysis based on each balance score at admission was used to examine its discriminative power to predict ambulatory independence and falls during hospitalization.

**Results:**

The mean (standard deviation) change between admission and discharge for each balance scale was 4.4 (4.7) for the Mini-Balance Evaluation Systems Test and 8.3 (10.0) for the Berg Balance Scale, with standard response means, a measure of responsiveness of 0.9 (large) and 0.8 (medium), respectively. Each balance score at admission almost equally predicted gait independence and fallers during hospitalization. On the contrary, only the distribution of scores on the Berg Balance Scale at discharge showed a ceiling effect, with 25 patients (43%) obtaining a perfect score.

**Discussion:**

The Mini-Balance Evaluation Systems Test may be useful as a balance measure for inpatients with subacute stroke because it is less susceptible to ceiling effects and more responsive than the Berg Balance Scale.

## 1 Introduction

Balance can be defined as the ability to maintain and restore the center of gravity line during continuous changes in the base of support through motor strategies relying on the integration of environmental sensory information (1). Patients with stroke are prone to balance problems, which not only increase the risk of falling (2, 3) but also decrease mobility (4), affect activities of daily living (5), reduce quality of life (6, 7), and limit social participation (8, 9). Therefore, it is necessary to identify balance disorders and related changes accurately.

Among the various clinical scales for assessing balance ability, the Berg Balance Scale (BBS) (10) is the gold standard and is the most widely used scale in clinical practice (11, 12). However, the BBS does not include dynamic balance components such as reactive postural control and dynamic gait. Therefore, the Balance Evaluation System Test was developed to assess dynamic balance (13). A shortened version of the test, the Mini-Balance Evaluation System Test (Mini-BESTest) (14), has recently been published, which consists of four balance sections (anticipatory postural adjustments, postural responses, sensory orientation, and stability in gait). Its reliability and validity have been demonstrated in various diseases and different countries (15–17) and it is widely used for balance assessment in clinical situations (18–20).

The length of hospitalization covered by insurance at Japanese convalescent rehabilitation wards (21) is up to 150 and 180 days in cases of stroke and stroke with severe disability or cognitive impairment, respectively. In addition, most patients are admitted to this ward within an average of 31.5 days after stroke onset. In other words, it is a period of marked improvement in function and ability for patients with stroke (22–25). There is a lack of sufficient discussion on whether the Mini-BESTest or the BBS should be used to assess balance in actual clinical settings during these relatively long periods of hospitalization and significant functional and functional capacity changes.

The purpose of this study was to examine the longitudinal association between the Mini-BESTest and BBS in inpatients with subacute stroke and to test the utility and applicability of each balance score in clinical practice.

## 2 Materials and methods

### 2.1 Study design

This prospective cohort study evaluated the floor and ceiling effect, validity (concurrent, convergent, and discrimination), and responsiveness of the Mini-BESTest and BBS in patients with subacute stroke. The study protocol was approved by the Institutional Review Board of Tokyo Bay Rehabilitation Hospital, Japan (approval number 85), and was registered before the study was conducted (UMIN000012875). This study was conducted as per the Declaration of Helsinki (revised in 2013), and all patients provided written informed consent before study enrollment. The study results were reported according to the Strengthening Reporting of Observational Studies in Epidemiology reporting guidelines.

### 2.2 Study setting and participants

The study was conducted at the Tokyo Bay Rehabilitation Hospital, which has convalescent rehabilitation wards (21). All patients with stroke who were admitted to the hospital between February 2014 and December 2014 were consecutively screened. The inclusion criteria for this study were as follows: patients with first-ever stroke hemiplegia, the ability to follow three-step commands on the Mini-Mental State Examination (MMSE) item (26, 27), and understanding the Mini-BESTest task. The exclusion criteria were: comorbidities affecting balance function, history of osteoarticular disease, and significant deformity or pain.

### 2.3 Data collection

The principal investigator, a physical therapist, reviewed medical records within 3 days of patient admission. Participants were assessed at baseline (within 1 week of admission) and at discharge with the Mini-BESTest and BBS for balance function, the Stroke Impairment Assessment Set (SIAS) motor and sensory items for motor/sensory function, and the Functional Independence Measure (FIM) for activities of daily living, respectively. The Mini-BESTest, BBS, and SIAS were performed by a well-trained physical therapist, and the FIM by the nurse.

#### 2.3.1 Mini-Balance Evaluation Systems Test

The Mini-BESTest, a 14-item clinical test (Table 1), covers four components of dynamic balance (anticipatory postural adjustments, postural responses, sensory orientation, and stability in gait) (14). Each item is scored from 0 (unable or requiring help) to 2 (normal), with a maximum score of 28 points. A greater score indicates better balance. This test has had excellent intra-examiner reliability and validity (28), and its advantage is the small ceiling effect in patients with stroke (28). In this study, the Japanese version of the Mini-BESTest (16) was used for evaluation. This Japanese version, like the original Mini-BESTest, has been shown to be reliable and valid in stroke patients (17).

**Table 1:** Mini-Balance Evaluation Systems Test and Berg Balance Scale components and scores.

Mini-Balance Evaluation Systems Test	
Anticipatory postural adjustments	Score range
1. Sit to stand	0–2
2. Rise to toes	0–2
3. Stand on one leg	0–2
Postural responses	
4. Compensatory stepping correction-forward	0–2
5. Compensatory stepping correction-backward	0–2
6. Compensatory stepping correction-lateral	0–2
Sensory orientation	
7. Stance (feet together); eyes open, firm surface	0–2
8. Stance (feet together); eyes closed, foam surface	0–2
9. Incline-eyes closed	0–2
Dynamic gait	
10. Change in gait speed	0–2
11. Walk with head turns-horizontal	0–2
12. Walk with pivot turns	0–2
13. Step over obstacles	0–2
14. Timed up & go with dual task	0–2
Berg Balance Scale	
Components	Score range
1. Sitting to standing	0–4
2. Standing unsupported	0–4
3. Sitting unsupported	0–4
4. Standing to sitting	0–4
5. Transfers	0–4
6. Standing with eyes closed	0–4
7. Standing with feet together	0–4
8. Reaching forward with outstretched arm	0–4
9. Retrieving object from floor	0–4
10. Turning to look behind	0–4
11. Turning 360 degrees	0–4
12. Placing alternate foot on stool	0–4
13. Standing with one foot in front	0–4
14. Standing on one foot	0–4

#### 2.3.2 Berg Balance Scale

The BBS (29) consists of 14 items (Table 1), including static and dynamic tasks of varying difficulty. Each item is scored on a scale of 0–4, with a maximum test score of 56. Higher scores indicate greater balance ability. The test has excellent reliability and validity in patients with stroke (30, 31) and is excellent for determining falls (31) and gait speed (32) in chronic patients with stroke.

#### 2.3.3 Stroke Impairment Assessment Set

The severity of motor paresis was assessed using the total SIAS-motor score (33). The upper limb motor score consists of two tests of the proximal and distal joints of the upper limb (0–12 points), and the lower limb motor score consists of 3 tests of the hip, knee, and ankle (0–15 points). The higher the score, the better the function. Its reliability and validity have been confirmed in patients with stroke (33, 34).

#### 2.3.4 Functional Independence Measure

Activities of daily living were measured using the FIM, which was assessed by trained nurses. The FIM consists of 13 motor subscales (FIM motor) and five cognitive subscales (FIM cognitive). Items on the scale are rated on a 7-point scale, with 1 representing complete dependence and 7 representing complete independence (35, 36). The reliability and validity of this measure have been confirmed in patients with stroke (37).

#### 2.3.5 Other variables

Characteristic information of the participants, such as age, sex, time since onset, and lesions, as well as the MMSE (26, 27) score as a screening for cognitive impairment, was collected from the patients’ medical records. Data on falls that occurred during hospitalization were collected from incident reports.

### 2.4 Analyses

All data analyses were performed using STATA/BE 17 (StataCorp., College Station, Texas, USA). *P*-values less than 0.05 were considered statistically significant.

#### 2.4.1 Validity

Concurrent validity was examined using the correlation between the Mini-BESTest and the BBS. The convergent validity was assessed using Spearman's correlation coefficient (rho) between each balance score and the FIM total score and SIAS-motor upper and lower limb total scores. The values for the correlation coefficient (rho) are classified with correlation values of: 0.00–0.10 (negligible); 0.11–0.39 (weak correlation); 0.49–0.69 (moderate correlation); 0.70–0.89 (strong correlation); and 0.90–1.00 (very strong correlation) (38). Cronbach alphas for Mini-BESTest and BBS were calculated as internal consistency. Alpha coefficients ranged from 0 to 1, and greater than 0.7 indicates good reliability (39, 40).

#### 2.4.2 Distribution of the scores

The distribution of scores for Mini-BESTest and BBS at admission and discharge was confirmed using scatter plots and histograms. The percentages of participants with the lowest and highest scores were also examined to further evaluate the floor and ceiling effects. The floor or ceiling effect was determined when at least 20% of the participants reached a specific score for the upper and lower limits (41).

#### 2.4.3 Ability to identify status

Receiver Operating Characteristic (ROC) analysis was used to assess the ability (predictive validity) to discriminate between ambulatory independence and non-independence at discharge from the Mini-BESTest and BBS admission scores. Gait independence was defined as follows: 1–5 FIM walk score at discharge as non-independent and 6 or more as independent. In addition, the ability to discriminate between Mini-BESTest and BBS scores at admission for falls that occurred during the observation period was examined using ROC analysis, in which the Youden index was used to calculate the cut-off value, and the area under the curve (AUC) and 95% confidence interval were determined (42). The AUC was interpreted as follows: AUC = 0.5 indicated no discrimination, 0.7 ≤ AUC < 0.8 indicated acceptable discrimination, 0.8 ≤ AUC < 0.9 indicated excellent discrimination, and AUC ≥ 0.9 indicated outstanding discrimination (43).

#### 2.4.4 Responsiveness

The values at admission and discharge for each balance score were compared using Wilcoxon's signed rank sum test, and the standardized response mean (SRM) was calculated. The SRM, a type of effect size, is defined as the mean change in score divided by the standard deviation of the changed score (44). According to Cohen's criteria (45), an SRM greater than 0.8 is defined as large, 0.5–0.8 as medium, and 0.2–0.5 as small.

## 3 Results

**Figure 1:**
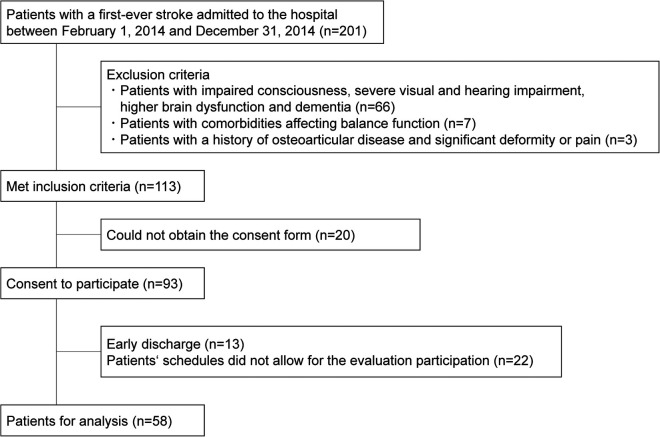
Flowchart of the patient selection process.

Among 201 consecutively enrolled patients with stroke, 98 met the selection criteria and gave consent, of which 35 were excluded for not being able to complete all assessments due to early discharge or other reasons, and 58 were ultimately included in the analysis (Figure 1).

Table 2 summarizes patient characteristics at admission. Of all participants (*n* = 58), 40 (69.0%) were male, the mean (standard deviation) age was 63.7 (13.2) years, the mean (standard deviation) days from stroke onset was 35.4 (13.0) days, the mean (standard deviation) hospital days was 74.6 (40.0) days, and the median (interquartile range) MMSE was 27.0 (23.0–29.0). The median (interquartile range) score for each balance scale at admission was 17.0 (10.0–24.0) for Mini-BESTest and 48.5 (35.0–54.0) for BBS.

**Table 2:** Characteristics of the participants at admission (*n* = 58).

Variables	
Sex, male/female, *n*, (%)	40/18 (69.0/31.0)
Age, years, mean (SD)	63.7 (13.2)
Days from stroke onset, mean (SD)	35.4 (13.0)
Number of days of hospitalization, mean (SD)	74.6 (40.0)
Types of stroke, infarction/hemorrhage/subarachnoid hemorrhage	34/20/4
Location of stroke, anterior cerebral artery/middle cerebral artery/posterior cerebral artery/basilar artery	5/34/6/13
Affected side, right/left/bilateral, *n*	30/26/2
Mini-Mental State Examination, median (IQR)	27.0 (23.0–29.0)
Mini-Balance Evaluation Systems Test, median (IQR)	17.0 (10.0–24.0)
Berg Balance Scale, median (IQR)	48.5 (35.0–54.0)
Stroke Impairment Assessment Set	
Upper limb motor function total score, median (IQR)	8.0 (5.0–10.0)
Lower limb motor function total score, median (IQR)	13.0 (12.0–15.0)
Upper limb sensory function total score, median (IQR)	5.5 (5.0–6.0)
Lower limb sensory function total score, median (IQR)	5.0 (4.0–6.0)
Functional Independence Measure	
Total score, median (IQR)	94.0 (79.0–106.0)
Motor item total score, median (IQR)	65.0 (53.0–75.0)
Cognitive item total score, median (IQR)	31.0 (25.0–34.0)

SD, standard deviation; IQR, interquartile range.

**Figure 2:**
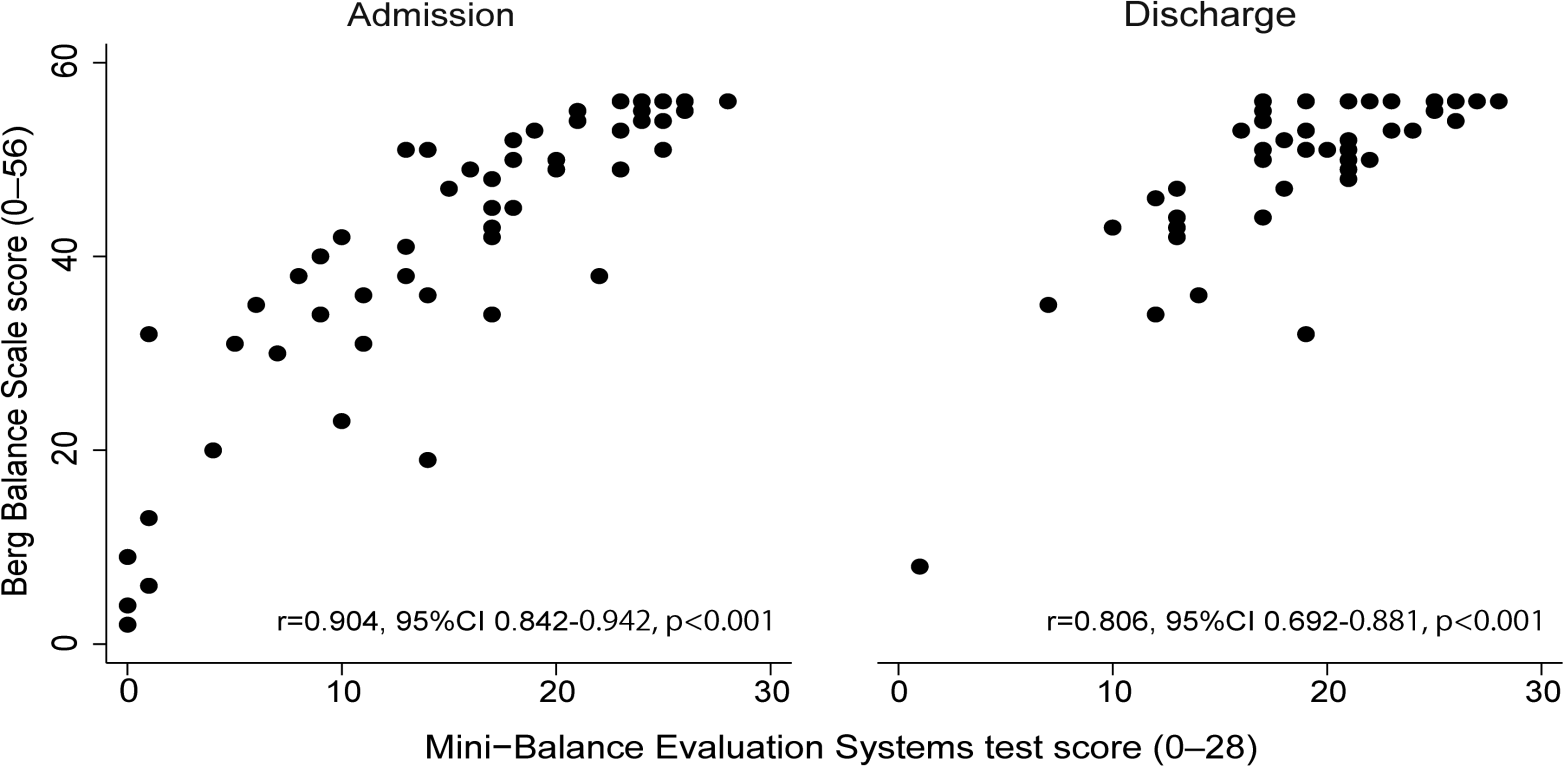
Relationship between Mini-Balance Evaluation Systems Test and Berg Balance Scale at admission and discharge.

Figure 2 shows the concurrent validity of each balance scale. The Mini-BESTest and BBS showed a very high correlation at admission (*r* = 0.90, 95% CI 0.84–0.94, *p* < 0.001) and a strong correlation at discharge (*r* = 0.81, 95% CI 0.69–0.88, *p* < 0.001). The alpha coefficients for the Mini-BESTest and BBS admission and discharge scores were 0.87 and 0.79, respectively, indicating good internal consistency for both. Table 3 shows the convergent validity of each balance scale with the FIM total score, a measure of activities of daily living, and the SIAS-motor upper and lower extremity total score, which indicates the severity of paralysis. All scores showed weak to moderate correlations.

**Table 3:** Convergent validity of the balance scale with FIM and SIAS-m.

		FIM total			SIAS motor total		
		rho	95%CI	*P* value	rho	95%CI	*P* value
Admission	BBS	0.60 (moderate)	0.40–0.74	<0.001	0.60 (moderate)	0.40–0.74	<0.001
	Mini-BESTest	0.55 (moderate)	0.34–0.71	<0.001	0.58 (moderate)	0.38–0.73	<0.001
Discharge	BBS	0.54 (moderate)	0.33–0.71	<0.001	0.51 (moderate)	0.29–0.68	<0.001
	Mini-BESTest	0.61 (moderate)	0.42–0.75	<0.001	0.35 (weak)	0.11–0.56	0.006

FIM, functional independence measure; BBS, Berg Balance Scale; Mini-BESTest, Mini Balance Evaluation Systems Test; SIAS, stroke impairment assessment set.

**Figure 3:**
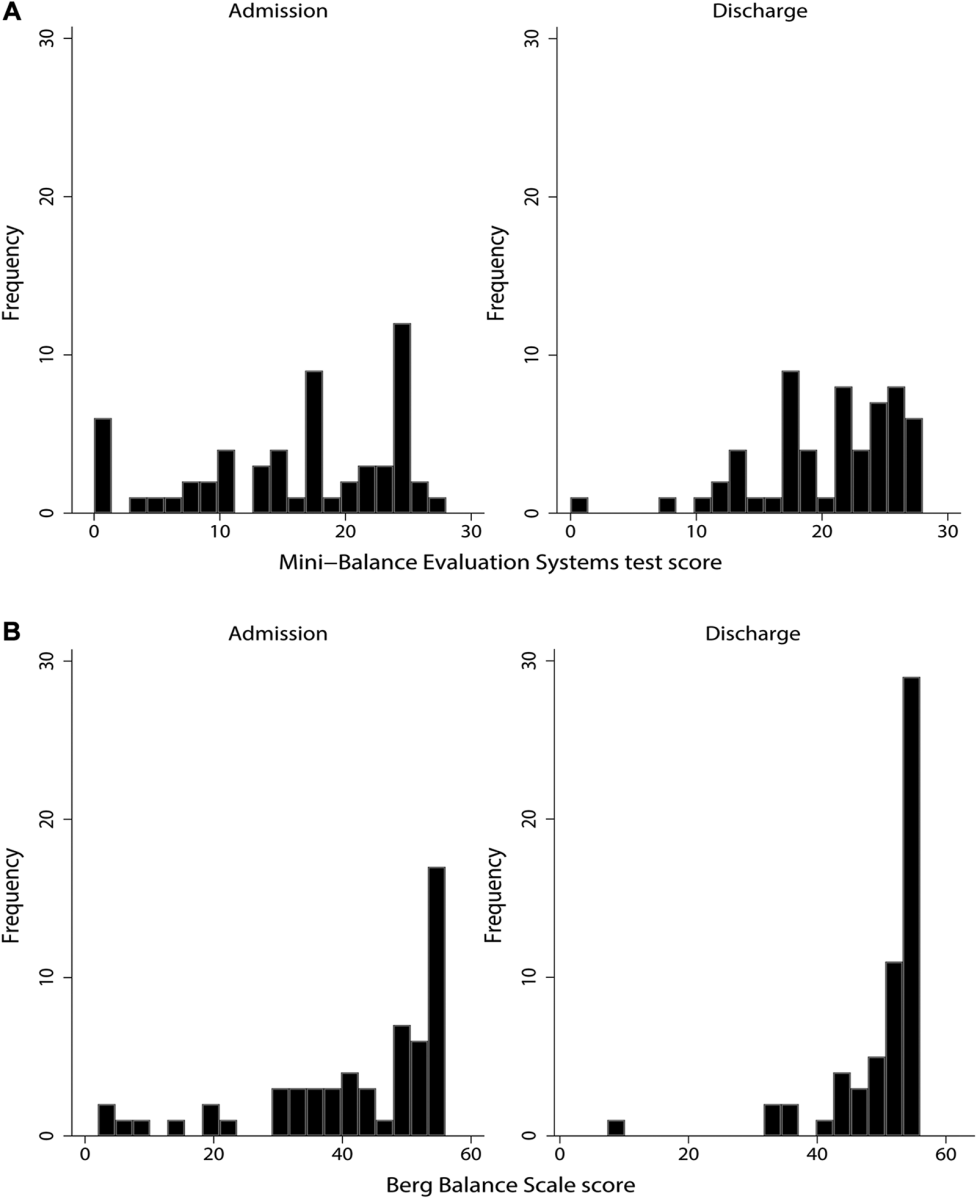
Distribution of Mini-Balance Evaluation Systems Test and Berg Balance Scale scores.

Figure 3 shows histograms of the distribution of scores for each balance scale. Skewness, an index of distribution asymmetry, was 0.074 at admission and 0.005 at discharge for Mini-BESTest (Figure 3A) and <0.001 at both admission and discharge for BBS (Figure 3B), indicating that BBS had a skewed distribution toward higher scores compared to Mini-BESTest. The lowest score and the proportion of patients with the lowest score were 0 for 3 patients (5.2%) at admission and 1 for 1 patient (1.7%) at discharge for Mini-BESTest, and 2 for 1 patient (1.7%) at admission and 1 for 1 patient (1.7%) for 8 at discharge for BBS. The percentage of perfect scores was 1.7% for 1 patient at admission and 5.2% for 3 patients at discharge for Mini-BESTest, 19% for 11 patients at admission, and 43.1% for 25 patients at discharge for BBS, indicating a ceiling effect for BBS.

**Figure 4:**
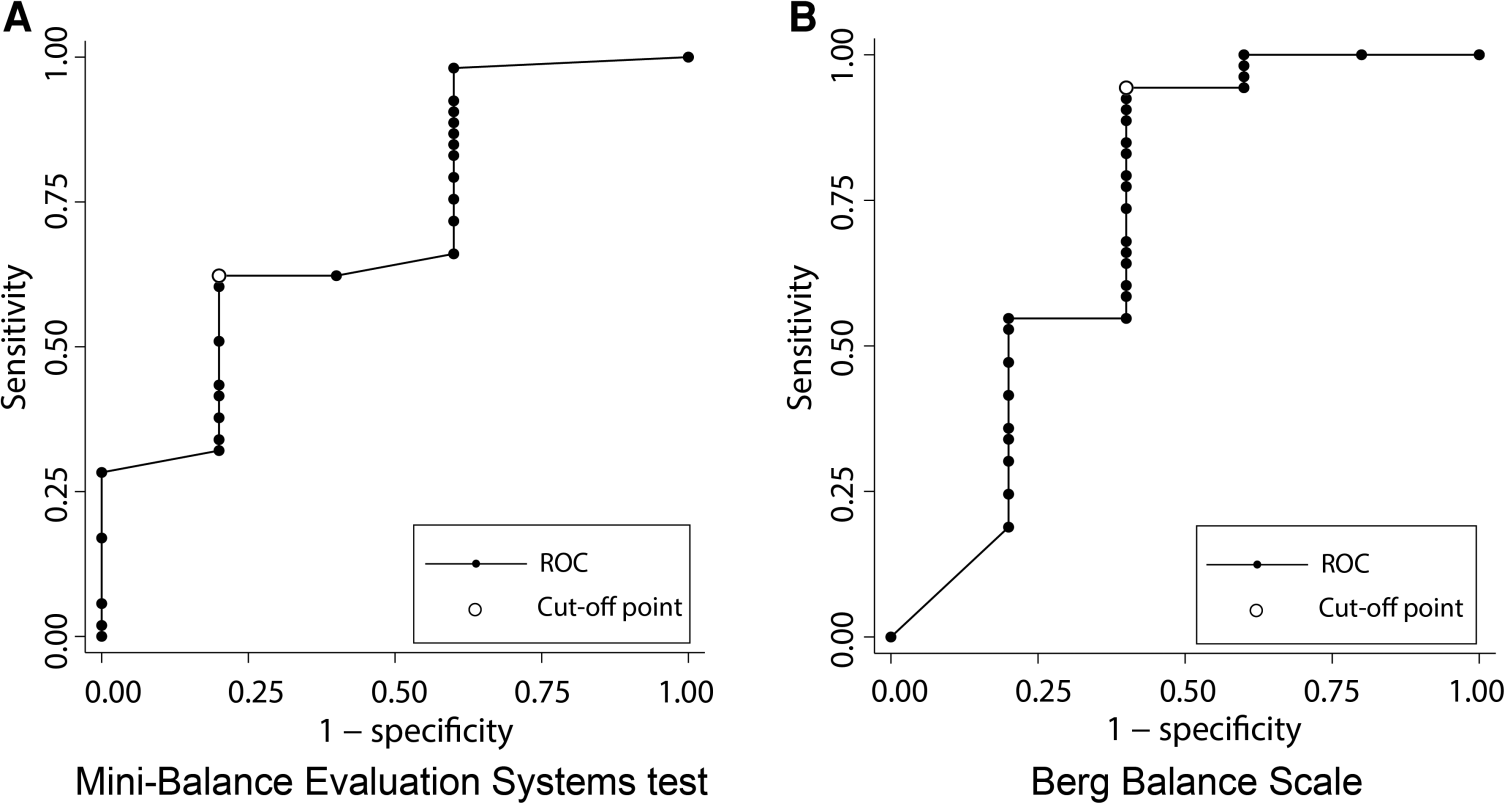
Receiver operating characteristic (ROC) curve plots of Mini-Balance Evaluation Systems Test and Berg Balance Scale for classifying gait independence during hospitalization. (**A**) ROC curve for gait independence discrimination in Mini-Balance Evaluation Systems Test. AUC = 0.74 (95% CI 0.45–0.95), cut-off point of 15 (sensitivity 62%, specificity 60%). (**B**) ROC curve for gait independence discrimination in Berg Balance Scale. AUC = 0.72 (95% CI 0.37–1.00), cut-off point of 19 (sensitivity 94%, specificity 40%).

**Figure 5:**
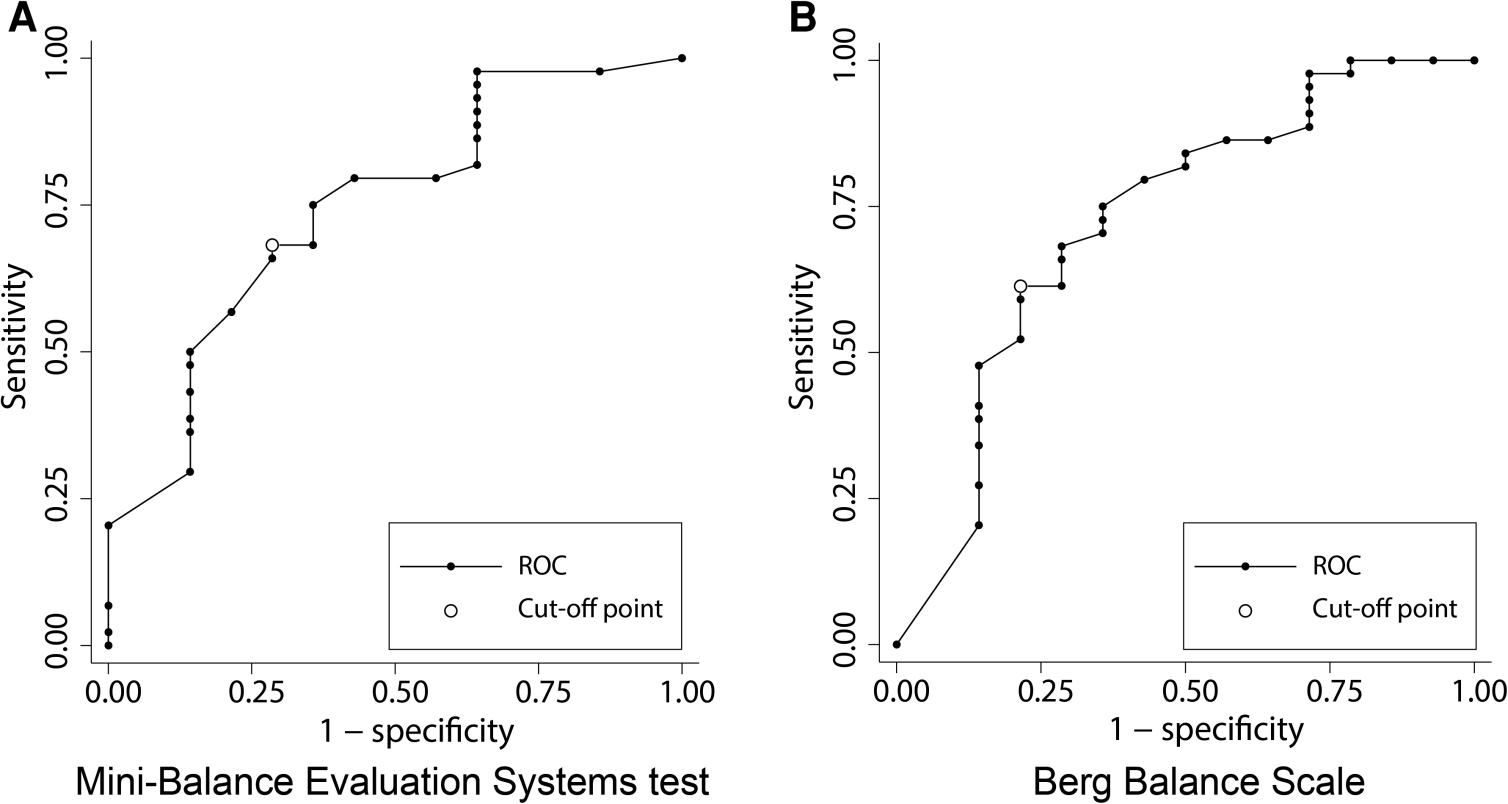
Receiver operating characteristic (ROC) curve plots of Mini-Balance Evaluation Systems Test and Berg Balance Scale for classifying the presence or absence of falls during hospitalization. (**A**) ROC curve for discrimination of fallers in Mini-Balance Evaluation Systems Test. AUC = 0.74 (95% CI 0.59–0.90), cut-off point of 15 (sensitivity 68%, specificity 64%). (**B**) ROC curve for discrimination of fallers in Berg Balance Scale. AUC = 0.73 (95% CI 0.55–0.90), cut-off point of 47 (sensitivity 91%, specificity 71%).

Figure 4 shows the results of the ROC analysis performed to discriminate gait independence at discharge from the admission scores of each balance scale. The AUC for Mini-BESTest was 0.74 (95% CI 0.45–0.97) with a cut-off point of 15 (sensitivity 62%, specificity 60%), and the AUC for BBS was 0.72 (95% CI 0.37–1.00) with a cut-off point of 19 (sensitivity 94%, specificity 40%), both acceptable predictors of gait independence. Figure 5 shows the predictive validity of falls occurring during the observation period using the admission scores for each balance scale. The AUC for Mini-BESTest was 0.74 (95% CI 0.59–0.90) with a cut-off point of 15 (sensitivity 68%, specificity 64%) and the AUC for BBS was 0.73 (95% CI 0.55–0.90) with a cut-off point of 47 (sensitivity 61%, specificity 71%), both acceptable predictors of fallers.

The mean (standard deviation) score for each balance scale was 16.0 (8.1) on admission and 20.4 (5.8) on discharge for Mini-BESTest and 42.3 (14.8) on admission and 50.6 (8.4) on discharge for BBS, respectively, showing significant changes (*p* < 0.001). The mean change (standard deviation) at admission and discharge for each balance scale was 4.4 (4.7) for Mini-BESTest and 8.3 (10.0) for BBS, with an SRM of 0.9 (large) for Mini-BESTest and 0.8 (medium) for BBS.

## 4 Discussion

This study demonstrated an association between the Mini-BESTest and changes in BBS over time in patients hospitalized with subacute stroke. The validity of the Mini-BESTest in subacute stroke inpatients was found to be similar to that of the BBS, which is the gold standard for balance assessment, with similar levels of walking independence and fall discrimination. Furthermore, compared to the BBS, the Mini-BESTest was less sensitive to ceiling effects and superior in responsiveness.

The BBS is one of the most commonly used clinical assessments of balance in patients with stroke (46, 47). In this study, the Mini-BESTest and BBS had excellent internal consistency, and a strong correlation was found between them, supporting concurrent validity. In addition, the FIM, a measure of activities of daily living, and the SIAS-m, a measure of paralysis severity, correlated moderately or better with their respective balance scales, confirming convergent validity. Furthermore, both Mini-BESTest and BBS showed acceptable discriminability in predicting gait at discharge and fallers during the observation period using each balance scale at admission. The Mini-BESTest sections (anticipatory postural adjustment, postural response, sensory orientation, and stability of gait) indicate the possibility of assessing various elements of balance function, which are not examined in the BBS. In fact, a previous study using the Mini-BESTest as an outcome showed that certain balance interventions selectively improved reactive postural control (20). Therefore, the Mini-BESTest could provide a more detailed assessment of the problematic parts of a patient's balance function and determine those for which the intervention was effective; hence, it could have high clinical applicability.

In the longitudinal course of the study, a ceiling effect was observed with 25 (43.1%) participants in the BBS compared to 3 (5.2%) in the Mini-BESTest, who reached their highest score at discharge on each balance scale. These results were similar to those of a cross-sectional study in patients with subacute stroke (17). The Mini-BESTest consists of a relatively broad spectrum of parameters ranging from low to high difficulty in dynamic balance, such as anticipatory postural adjustment, postural response, sensory orientation, and stability of gait. On the contrary, the BBS consists of items with low difficulty, such as static balance, which includes standing with eyes closed and left-right swinging movements. Against this background, the Mini-BESTest has less of a ceiling effect compared to the BBS, even in relatively well-functioning patients with stroke, suggesting that it may adequately assess changes in longitudinal balance function in these patients. This result could also be the reason for the Mini-BESTest to be more responsive than the BBS in the present study.

In general, patients with subacute stroke admitted to rehabilitation units are also in a period of marked improvement in function and ability (22–25). Therefore, it is desirable to use an assessment battery that is less susceptible to ceiling effects and more responsive to patient balance assessments. In this study, the Mini-BESTest was less susceptible to ceiling effects and more responsive than the BBS. It is clinically significant that Mini-BESTest was shown to be desirable for balance assessment in stroke patients hospitalized for relatively long durations.

A limitation of this study is that uptake was only about 30% of the total for consecutive cases admitted during the period. Therefore, it is possible that severe stroke patients were not included. Severe stroke patients in previous studies were defined as having a SIAS-m total of 5 or less points in the lower extremities (48). The patients included in this study had a median lower extremity SIAS-m total score of 8 (interquartile range 5.3–10.0) and may have been patients with moderate stroke. In a previous study, Mini-BESTest was found to have a floor effect, although it was less sensitive to ceiling effects (49). No floor effect was found in this study; however, the exclusion of severe cases from the study based on our inclusion criteria might have affected this finding. In addition, caution must be exercised in generalizing the results because the study was conducted at a single institution and in a country with a health care system that has relatively long hospital stays.

## 5 Conclusions

These results suggest that the Mini-BESTest, like the BBS, may be a measure that can reflect balance ability in recovering stroke patients. The Mini-BESTest is less susceptible to ceiling effects than the BBS, is more responsive, and can be assessed by balance components, indicating that it may have greater clinical applicability.

## Data Availability

The raw data supporting the conclusions of this article will be made available by the authors, without undue reservation.
